# Plastic flow anisotropy drives shear fracture

**DOI:** 10.1038/s41598-018-38437-y

**Published:** 2019-02-05

**Authors:** A. Amine Benzerga, Nithin Thomas, Joshua S. Herrington

**Affiliations:** 1 Texas A&M University, Department of Aerospace Egineering, College Station, TX 77843-3141 USA; 2 Texas A&M University, Department of Materials Science & Egineering, College Station, TX 77843-3141 USA

## Abstract

Fracture of initially crack-free bodies often occurs due to plastic instabilities known as shear bands. Previous computer simulations advanced a myriad of mechanisms to rationalize shear banding. However, they were restricted to planar geometries. We investigate the relevance of anisotropic plasticity by picking an axisymmetric tensile test rig, in which shear localization is rarely observed. The three-dimensional finite-element simulations of shear banding in this type of specimens are the first of their kind. The micromechanical modeling covers a range of competing mechanisms believed to be responsible for such failure. We show that anisotropic plasticity can effectively trigger shear bands thereby causing failure of ductile solids. Our results enable shear fracture to be rationalized in ductile rocks and mitigated against in designing advanced materials.

## Introduction

Fracture of initially crack-free bodies occurs by internal damage accumulation or by shear banding. The latter is one of the most deleterious failure processes^1^. Shear fractures cause enormous amounts of material and energy waste per annum, with severe economical, industrial and environmental impact. As a sudden, unstable phenomenon, shear failure is difficult to study experimentally. The phenomenon broadly occurs in metals^2–4^, polymers^5,6^, metallic glasses^7,8^, and in geologic and granular materials^9–12^. Despite considerable progress, basic understanding of what causes shear fractures across so many solids remains elusive. Predictive simulations are essential to understanding this complex phenomenon.

Previous analyses and computer simulations of shear band formation have suggested various mechanisms. These include pressure-sensitivity and dilatancy^13^, geometric or textural softening^3,14,15^, thermal softening^16^, internal friction or non-associative flow^7^, strain softening^17,18^, often porosity-induced^19,20^, and plastic anisotropy^20,21^. However, all of the above analyses (see Pineau *et al*.^22^ for a review) were restricted to planar geometries, which are notorious for being conducive to shear banding^23^. Furthermore, the orientation of shear bands transcends microstructural and atomic-scale details, although these play a key role^22^. In crystalline materials, for instance, shear bands are not to be conflated with slip bands^3^.

**Figure 1:**
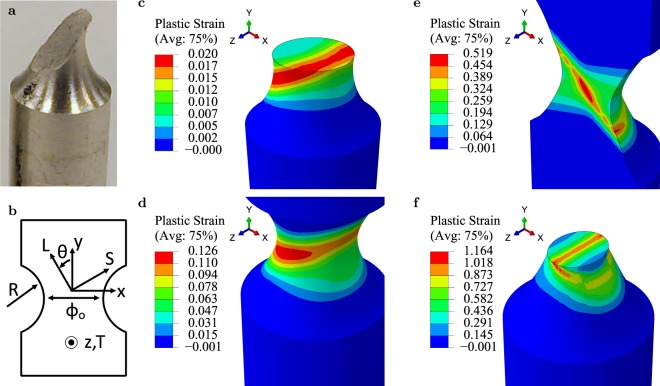
Shear-band induced shear fracture. (**a**) Example of shear fracture at ~0.2 overall nominal strain in a slightly misaligned ($\theta \le 10^\circ$) round notched bar of a magnesium alloy (courtesy of S. Basu and A. Benzerga). (**b**) Orientation of the material axes (L,T,S) with respect to the loading axes ($x,y,z$). (**c**–**f**) Snapshots of shear failure simulations in contours of effective plastic strain, $\bar{\varepsilon }$, showing the transient deformation band at incipience (**c**), and at limit load (**d**), the dilatational shear band (**e**), and the final shape of the bar from another perspective that highlights the non-planar shear band (**f**). Simulation parameters are ${h}_{{\rm{TS}}}/{h}_{{\rm{L}}}=2.333$, $\theta =30^\circ$, ${f}_{0}={10}^{-4}$, ${w}_{0}=1$, $E/{\sigma }_{0}=500$, $N=0.1$, and $R/{\varphi }_{0}=1$. See Supplementary Information, Movie S1.

Here, we investigate the interplay between various mechanisms by picking an axisymmetric tensile test rig, in which shear localization is not easy, Fig. 1a. Shear bands in such experiments have never been simulated before at any length or time scale. Fully three-dimensional simulations are carried out using a constitutive framework capable of describing the competition between porosity-induced softening and anisotropic plasticity in triggering shear failure; see Methods.

The wavelength associated with shear bands is generally far larger than can be simulated using atomistic methods. To investigate shear banding induced fracture, we carried out a large number of numerical experiments using a continuum micromechanics approach for porous material plasticity that extends an earlier formulation^24^ to anisotropy; see Methods. This approach has been applied to various anisotropic fracture problems^22,25^. In a ductile fracture context, it provides a consistent, coupled description of both macroscopic finite-deformation response and micro-scale material damage evolution. Damage is represented by microvoids, approximated as spheroids so that their shape and orientation, in addition to their volume fraction (porosity), are tracked space- and time-wise in a boundary value problem solution. Furthermore, the approach has the advantage of augmenting the initial or induced anisotropy due to void shape with the plastic flow anisotropy of the matrix. In contrast, previous finite-element based analyses have either neglected all forms of anisotropy^24,25^ or only considered aligned configurations whereby the principal axes of matrix anisotropy were parallel to the principal axes of loading^26^. Here, both the matrix anisotropy and misalignment are shown to play a crucial role. The broader significance of our findings will be discussed further in the context of rational design of advanced materials and fundamental understanding of naturally occurring phenomena.

## Results

We investigated the deformation of an initially crack-free round notched bar with an imposed uniform vertical displacement rate on the upper surface at $y=l/2$ and a fixed lower surface at $y=-\,l/2$. In most simulations, the top surface was free to move laterally in the *x*–*z* plane. The bar has a circumferential notch of radius *R* and minimum section initial diameter ${\varphi }_{0}$, Fig. 1b. Bar length *l* was taken to be large enough so as not to influence the results. All lateral surfaces were free of any tractions. The plastic anisotropy of the matrix is controlled by the ratio ${h}_{{\rm{TS}}}/{h}_{{\rm{L}}}$ where ${h}_{{\rm{TS}}}={h}_{{\rm{SL}}}$ and ${h}_{{\rm{L}}}={h}_{{\rm{T}}}={h}_{{\rm{LT}}}$ are anisotropy coefficients entering the yield condition, as described in Methods. In addition, we take ${h}_{{\rm{S}}}={h}_{{\rm{L}}}$ to focus on simple forms of transverse isotropy about the S axis. The material axes were oriented as shown in Fig. 1b with the angle *θ* measuring the misorientation with respect to the loading direction. We assume that the material contains a very small amount of initial porosity ${f}_{0}\le {10}^{-4}$ made up by spherical voids (aspect ratio ${w}_{0}=1$) but void shape changes and rotations are then induced by deformation. The effective material (matrix and voids) is not transversely isotropic at any stage of deformation. All simulations, the $\theta =0$ case included, were fully three-dimensional to account for the potential symmetry breaking induced by strain localization.

### Process of shear banding

**Figure 2:**
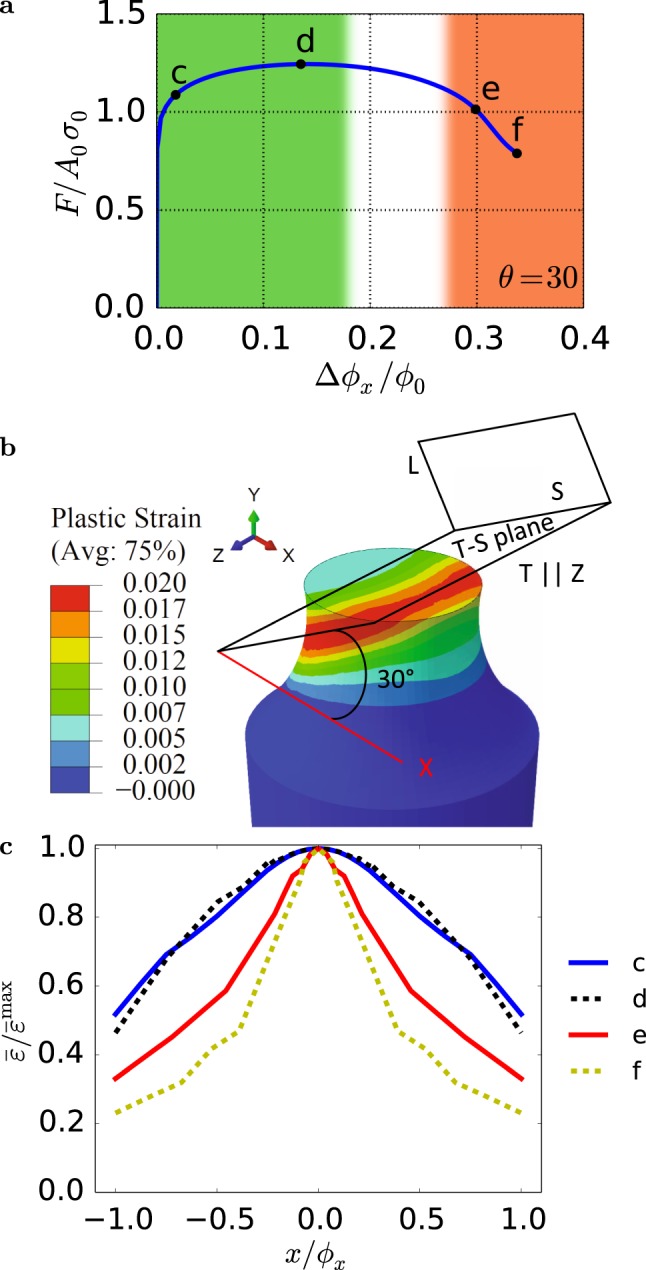
Instability development and growth. (**a**) Normalized load versus minimum-section diameter reduction with ${A}_{0}=\pi {\varphi }_{0}^{2}/4$ and *σ*_0_ a reference yield strength. Green and red regions depict the evolving transient and shear bands, respectively, while the white region depicts the transition between them. (**b**) Orientation of the transient deformation band. (**c**) Plots of effective plastic strain normalized by its maximum along a center line section parallel to *x* at $y=0$; for reference ${\bar{\varepsilon }}^{{\rm{\max }}}=0.017$ and 0.95 at stages c and f, respectively. In (**a** and **c**) labels c to f refer to the stages shown in Fig. 1.

An example of bar deformation leading to shear banding is shown in Fig. 1. The various stages c–f map onto the loading response of Fig. 2a. Various bar sections are selected for enhanced rendering of three-dimensional aspects. A transient deformation band forms in the early stages of loading (Fig. 1c) and persists until a limit load is reached (Fig. 1d). The transient band causes a perturbation, which gives way to a non-planar shear band (Fig. 1e), i.e., a band of concentrated plastic strain with elastic unloading taking place outside of it. A significant load drop accompanies shear band formation, Fig. 2a. The plastic instability subsequently develops by enhanced plastic strain concentration inside the band (compare stages e and f). In the absence of any rate-sensitivity or other localization limiters, the plastic strain in the band is determined by the finite-element size, which is taken to be representative of the mean void spacing.

### Transient versus shear bands

To gain further insight into the deformation process leading to shear banding, the orientation of the transient deformation band was analyzed, Fig. 2b. Because of initial axial symmetry, the choice of transverse axes *x* and *z* associated with the bar itself is arbitrary. However, for a given choice of material axes (L,T,S) with misorientation *θ* (Fig. 1a) the *z* axis is identified with material axis T. Hence, the angle between the *x* axis and the S orientation is also *θ* (=30° in the case analyzed here). Interestingly, the transient band is parallel to the T–S plane and its orientation is precisely 30° from the *x* axis, Fig. 2b. Its thickness is set by the notch induced strain gradients. For a value of the anisotropy ratio ${h}_{{\rm{TS}}}/{h}_{{\rm{L}}}$ larger than unity, the material is weaker in shear in principal planes containing the S axis than in any other plane. There are two such planes, T–S and L–S, the latter resolving a lower shear stress under the loading conditions considered. Thus, the incipient band (stage C in Fig. 2a) is akin a slip band in a crystal oriented for single slip, hence the term “deformation band”. This band is not a shear band in the sense that it does not result from an instability of any sort. It is transient because the ensuing deformation is affected by the complex stress state induced by the notch so that the band eventually disappears after the maximum load. In sum, the transient deformation band has an orientation that is determined by initial anisotropy and creates a perturbation that eventually triggers the instability at a later stage.

Strain concentration in the shear band is much higher than in the transient band. This is illustrated by taking line section plots in the bar’s mid-plane $y=0$, Fig. 2c. Because of plastic anisotropy, initially circular sections become oval (see Fig. 1f) so that ${\varphi }_{x}\ne {\varphi }_{z}$ at any stage but initially. The highest strain gradients are obtained along *x*, as shown in Fig. 2c where the effective plastic strain is normalized by its maximum along the line. Prior to shear banding (stages c–d), the strain concentration has a fairly constant gradient, which is set by the notch geometry. As inferred from the contour plots in Fig. 1, the maximum strain levels are much higher once the shear band has formed and continue to increase in magnitude and gradient. Unlike the transient deformation band, the shear band has an orientation that is unrelated to the material axes, Fig. 1e.

### Damage growth in the band

**Figure 3:**
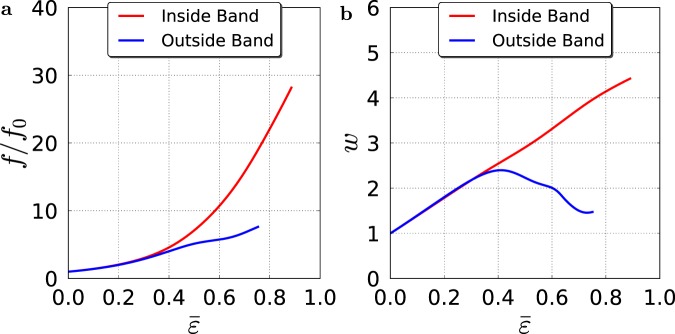
Porosity in the shear band and outside. (**a**) Porosity (normalized by its initial value) versus effective plastic strain, $\bar{\varepsilon }$, inside the shear band (center of bar) and a distance away from the band. (**b**) Void aspect ratio, *w*, versus $\bar{\varepsilon }$ at same locations.

The above deformation patterns are affected by competition between high hydrostatic stress levels at the center, which promote rapid porosity growth and further strain softening, and notch-induced strain concentrations at the notch root. Because the shear bands are dilational, faster void growth occurs therein post-localization, Fig. 3a. Outside the band, contraction occurs in its immediate vicinity due to high shear stresses in the *x*-*y* plane, in addition to normal stresses, and this leads to rotating the shrinking voids parallel to the band. Inside the band, on the other hand, the voids gradually rotate to align themselves with the band and further elongate, Fig. 3b, while increasing in volume until void coalescence occurs. Coalescence inside shear bands has recently been observed in metallic glasses^27^.

Material separation inside the shear band, which is not explicitly simulated here, would occur in the highly porous region once void coalescence is complete^28^. The porous region is contained within, but does not fully consume the shear band. Comparison between porosity contours on the outer surface of the bar and in meridian planes (see Movies S2 and S3) shows that the porous band has a complex three-dimensional shape that does not fully traverse the bar.

### Comparison with experiments

**Figure 4:**
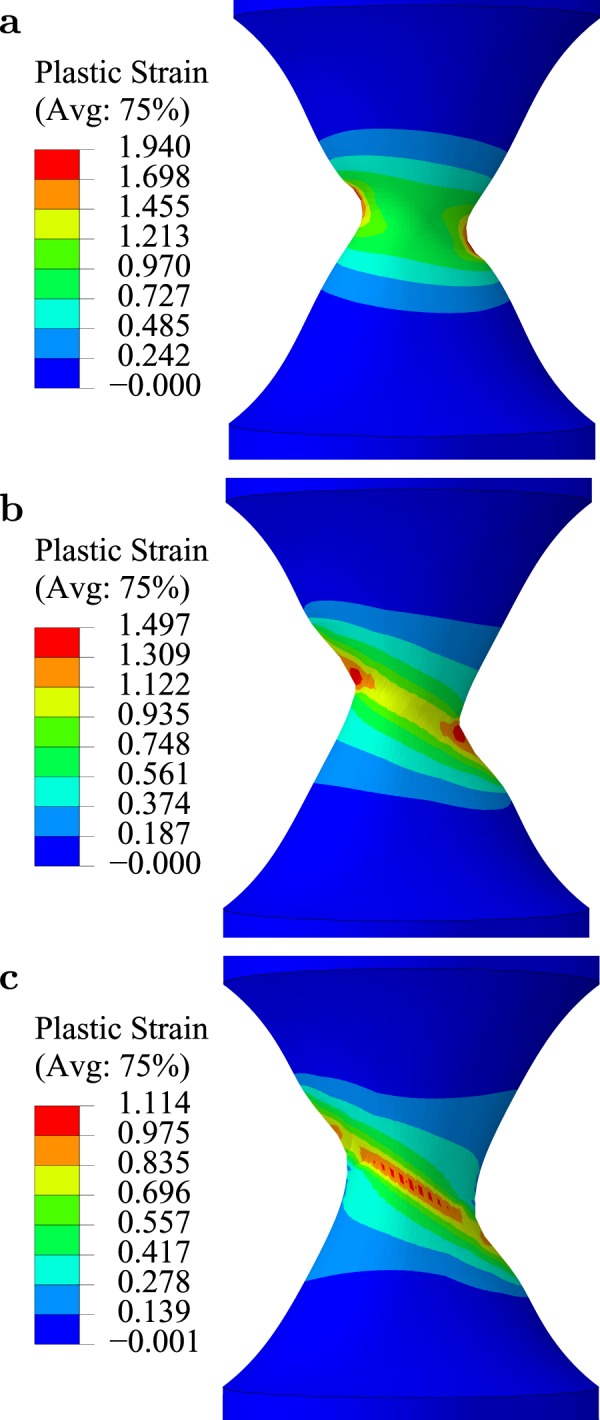
Contours of effective plastic strain for various misorientations. (**a**) Initial misorientation of $\theta =5^\circ$ (at ${\rm{\Delta }}{\varphi }_{x}/{\varphi }_{0}=0.40$). (**b**) Initial misorientation of $\theta =15^\circ$ (at ${\rm{\Delta }}{\varphi }_{x}/{\varphi }_{0}=0.37$). (**c**) Initial misorientation of $\theta =25^\circ$ (at ${\rm{\Delta }}{\varphi }_{x}/{\varphi }_{0}=0.34$). All other simulation parameters are as in Fig. 1.

In Figs 1–3, the initial misorientation was $\theta =30^\circ$ but additional calculations showed shear banding for smaller misorientations, Fig. 4. A remarkable finding is that, when void coalescence is turned off and shear banding initiates at the center of the bar, the orientation of the shear band is approximately the same regardless of the initial misorientation *θ*, Fig. 4b,c. There are exceptions corresponding to $\theta \le 10^\circ$ due to a propensity for shear band initiation at the free surfaces, as illustrated in Fig. 4a (compare with shear banding inside the bar as in Fig. 4b,c). The dual shear banding observed for lower misorientations is qualitatively consistent with some post-mortem experimental observations (Supplementary Fig. S2).

**Figure 5:**
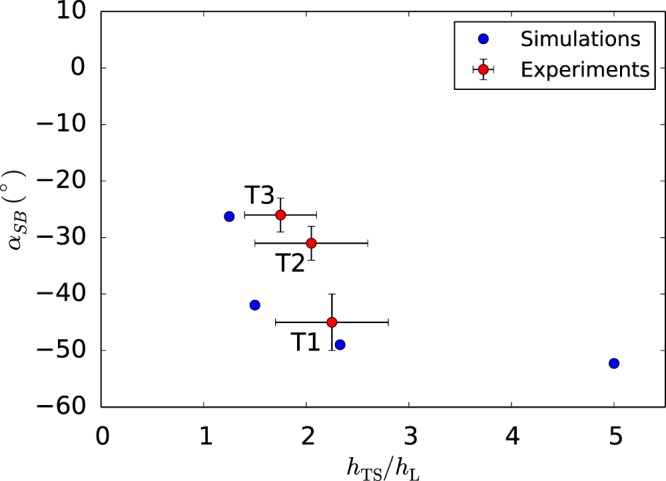
Effect of anisotropy on shear-band angles. Comparison of predicted shear band angles with experimental data in three magnesium alloys reproduced from ref.^29^ (textures T2 and T3) and Fig. 1a (texture T1) using the exact same geometry ($R/{\varphi }_{0}=1$). Predicted angles are evaluated at the core of the shear band for various values of the anisotropy ratio ${h}_{{\rm{TS}}}/{h}_{{\rm{L}}}$. Other simulation parameters are as in Fig. 1. Horizontal error bars account for estimated values of ${h}_{{\rm{TS}}}/{h}_{{\rm{L}}}$, given that the anisotropy of the alloys is more complex.

Furthermore, we have investigated the effect of the magnitude of plastic anisotropy, as measured by the single ratio ${h}_{{\rm{TS}}}/{h}_{{\rm{L}}}$, for fixed misorientation $\theta =30^\circ$. In particular, we extracted the final shear band angles, Fig. 5 (see Supplementary Fig. S1 for details). For an isotropic matrix, ${h}_{{\rm{TS}}}/{h}_{{\rm{L}}}=1$, no shear band was found to form, even if the effective material has induced anisotropy due to void shape changes. For ${h}_{{\rm{TS}}}/{h}_{{\rm{L}}} > 1$, the shear band angle is dependent on anisotropy (see Movies S4 and S5). Planes that are weak in shear with the highest resolved shear stress define the orientation of the transient band. Since the latter acts as a perturbation, both its orientation and strain level matter. In contrast, for ${h}_{{\rm{TS}}}/{h}_{{\rm{L}}} < 1$, the material is shear-resistant in S–L and S–T planes. A deformation band does not form until large strains but the specimen’s orientation is too stiff and void coalescence occurs before any localization (see Movies S6 and S7). The simulation results in Fig. 5 predict a strong effect of the magnitude of anisotropy, at fixed misorientation, on (i) the propensity for shear banding, and (ii) the shear band angles.

In Fig. 5, we qualitatively compare the model predictions for the shear band angle with experimental measurements of shear fractures (Fig. S2) in three polycrystalline magnesium alloys having the same chemical composition but different textures resulting in different net plastic anisotropies^29^. We carry out this comparison using *θ* = 30° in the simulations although misorientation angles in the experiments are less than 10°. This is done to emphasize that, while some initial misorientation is necessary for shear banding, the exact value of initial misorientation has a secondary effect on the shear band angle, as noted in Fig. 4. Any effects observed for $\theta  < 10^\circ$ are not intrinsic, but essentially structural, due to the interaction of incipient shear bands with free boundaries (Fig. 4a). Applying Hill’s criterion to the evolving anisotropy of magnesium alloys is questionable. On more accurate representations, the reader is referred to recent work by Kondori *et al*.^30^. However, Hill’s coefficients provide a practical basis for a qualitative assessment. Since the anisotropy of these materials is more complex, comparison is made by considering shear coefficients *h*_TS_ and *h*_SL_, which are believed to play a key role. The spread of values reported in Fig. 5 for the abscissa accounts for the fact that the above materials were not transversely isotropic (${h}_{{\rm{TS}}}\ne {h}_{{\rm{SL}}}$) so that the range of values is indicative of such differences (see Table S1). The spread on the ordinate indicates experimental scatter and standard error measurement. Remarkably, a range of measured values for the shear band orientation can be rationalized this way in spite of the uncertainty on anisotropy coefficients. Thus, in the experiments as well as the simulations, a variation in plastic anisotropy leads to a variation in the angle of shear fracture.

## Discussion

Shear failure is highly detrimental in both material fabrication and in service. Identifying the driving forces for its occurrence is therefore an important objective for mitigating its effects over a wide range of manufacturing processes and load bearing structures. Shear fractures are also widely reported in natural matter^10^.

As a strain localization phenomenon, shear banding corresponds to a bifurcation in the instantaneous response in rate-independent solids^23^ and to the unbounded growth of initial perturbations in rate-dependent ones^25,31^. In particular, analysis based on Rice’s general framework^23^ permits two key observations: (i) localization is impossible in a strain-hardening material; and (ii) axisymmetric stress states are extremely stiff against localization. Accordingly, experimental observations of shear fracture in round specimens of ductile materials are rare, Fig. 1a. However, the fact that both observations are contingent upon the plastic behavior being isotropic has been overlooked.

Conditions that are potentially sufficient for shear banding are well documented in the literature^22^. However, identifying the cause of shear fracture in a given instance is generally unknown for there is more than one condition met in a given material. For example, yield vertex effects inherent to crystal or rock plasticity cannot be separated from the anisotropy of crystallographic slip^32^. Likewise, in low-symmetry materials, such as hexagonal close packed metals, strength differential effects are concurrent with strong anisotropy at atomic and texture levels^33^. What makes previous research inconclusive in this regard, across the fields, is that all previous computer simulations of shear banding were carried out for plane geometries^3,14,15,18,19,21,25^. Such mechanical states are notorious for being conducive to shear banding^23^ and hence cannot discriminate among the various possible mechanisms of shear fracture. A discriminating outcome is the occurrence of shear failure in three-dimensional specimens, such as axially symmetric bars. This is because axisymmetric stress and strain states are much stiffer against localization^23^.

Here, we investigated circumstances under which shear fracture occurs in cylindrical geometries by means of simulations inspired by experiments initiated by Basu *et al*.^29^. The micromechanical modeling accounts for two sources of anisotropic response: morphological, due to void shape effects, and structural, due to the anisotropy of the matrix itself. As such, the model encompasses, as a very special case, the widely used isotropic Gurson model^24^, which has proven successful in capturing key physical phenomena of ductile failure^25^. The model also encompasses the anisotropic model of Benzerga *et al*.^26^, which was validated against quantitative macro- and micro-scale experimental measurements. It improves upon it through a coupling between the two sources of anisotropy thanks to homogenization from first principles and through the void rotation law; see Methods. This micromechanical modeling covers a range of competing mechanisms believed to be broadly responsible for shear failure. Among all factors that are known to be potentially destabilizing, we have considered porosity-induced softening and anisotropic plasticity. Contrary to prevalent understanding based on Rice’s analysis^23^, we predicted shear band formation (i) in nominally axisymmetric states and (ii) while the instantaneous response of the material was still hardening (Supplementary Fig. S4). We emphasize that these findings are consistent with Rice’s theory^23^, which is applicable to anisotropic solids. Specifically, we found that the shear band orientation depends on the magnitude of plastic anisotropy (Fig. 5), in keeping with experiments, but weakly on the initial misorientation (Fig. 4).

The simulation results hint at the complex interplay between anisotropy and stress state. The former characterizes the material whereas the latter is imposed, at least initially, via the axially symmetric geometry and the loading scheme. Here, plastic anisotropy forces a strain state that is different from the one that is mechanically imposed. In particular, the anisotropy-induced mechanical state is akin a plane strain state, which presumably increases the propensity for shear banding. Note that the corresponding overall stress along the *z* axis is not determined by the in-plane stresses, unlike in strict plane strain. Such interplay comes out as a natural outcome to a boundary value problem solution, which has to be fully three-dimensional.

In the analyses reported here, we focused on simple forms of matrix anisotropy. Additional results for full orthotropy may be found under Supplementary Fig. S3. Also, strong anisotropies due to void shape at high porosity levels remain to be explored as would arise, for example, in cellular and like materials. Even with relatively simple forms of anisotropy reduced to a single parameter, a new phenomenon was uncovered. In varying the amount of initial porosity *f*_0_, we have found that even with a vanishingly small *f*_0_ shear banding would occur but with a much delayed onset of fracture^34^. The key role of initial misorientation of the material axes indicates that other potentially destabilizing factors, heretofore thought to play a key role^3,7,13,14,16–19^, may not be essential. In addition, successful modeling of shear band angles (Fig. 5) is indicative of the physical mechanism of shear banding. Therefore, plastic anisotropy is generally established as the main factor driving shear fracture.

Our findings pave the way for a whole new paradigm for designing with, not against anisotropy. Indeed, for much of the past half century or so, efforts have been diligently directed at suppressing plastic anisotropy, e.g. by alloying or thermomechanical processing in metallic materials in order to improve ductility. Such efforts have largely failed in the context of magnesium alloys^29^. There is no scientific principle stating that isotropy is optimal. We have recently introduced the anisotropy-effect-on-ductility (AED) index and validated its predictive capability against experiments^29^. The computations reported here show that for robust anisotropy engineering, one must account for a tradeoff between the AED index when fracture is damage-limited and another anisotropy-dependent index, yet to be developed, representing the propensity for shear banding. At present, a fully analytic expression of the latter index is not available given the complexity of the problem. However, calculations such as those reported here will help in developing numerical failure maps in a highly dimensional parameter space that would enable rational design of anisotropy for enhancing the performance of advanced materials. With parallel developments in atomic-scale understanding of what governs anisotropic plasticity and ductility^35–37^, the type of microscopic structural parameters to be manipulated will be further elucidated based on critical macroscopic indicators such as those developed here.

Anisotropy is ubiquitous in engineered and natural matter alike^7,10^ by the very natural formation or material synthesis processes. On the other hand, voids are observed to mediate failure in a variety of materials, from polycrystalline metals^22^ and metallic glasses^27^ to semi-crystalline polymers^38^ and geologic materials^39–41^. Our findings provide fundamental insight into shear fractures occurring in ductile deformed rocks^10^ where the interplay between void formation^39,40^, void collapse and coalescence^41^ and anisotropic plasticity must play a fundamental role in the development of shear bands and shear fractures in mid and lower crust rocks. Specifically, it is the anisotropic plasticity that emerges as key to driving shear fractures with porosity playing a secondary role.

## Methods

We use a constitutive framework capable of describing the competition between the micromechanics of damage accumulation and shear failure. Shear band formation was a natural outcome of simulations using a micromechanical rate-independent constitutive relation of a porous material. Voids are represented as spheroids with axis ${{\boldsymbol{n}}}^{\mathrm{(3)}}$. The yield function for the effective medium (matrix and pores) is given by^42,43^

1 $$ {\mathcal F} (\sigma )=C\frac{{\mathscr{Q}}(\,\sigma \,)}{{\bar{\sigma }}^{2}}+2(g+1)(g+f)\,\cosh \,(\kappa \frac{ {\mathcal L} (\sigma )}{\bar{\sigma }})-{(g+1)}^{2}-{(g+f)}^{2}$$

where $\bar{\sigma }$ is the flow strength of the matrix in some reference direction, while ${\mathscr{Q}}$ and ${\mathcal L}$ are quadratic and linear forms of the stress respectively given by ${\mathscr{Q}}(\sigma )=3/2\sigma :{\mathbb{H}}:\sigma$ and ${\mathcal L} (\sigma )=\sigma :X$. Here, ${\mathbb{H}}$ reduces to the plastic anisotropy tensor of the matrix, , if voids are prolate but, for oblate ones, it contains a porosity dependent term involving dyadic products between a constant tensor ${\mathscr{Q}}=-\,\frac{1}{2}({{\boldsymbol{n}}}^{(1)}\otimes {{\boldsymbol{n}}}^{(1)}+{{\boldsymbol{n}}}^{(2)}\otimes {{\boldsymbol{n}}}^{(2)})+{{\boldsymbol{n}}}^{(3)}\otimes {{\boldsymbol{n}}}^{(3)}$, and $X={\alpha }_{2}({{\boldsymbol{n}}}^{\mathrm{(1)}}\otimes {{\boldsymbol{n}}}^{\mathrm{(1)}}+{{\boldsymbol{n}}}^{\mathrm{(2)}}\otimes {{\boldsymbol{n}}}^{\mathrm{(2)}})+\mathrm{(1}-2{\alpha }_{2}){{\boldsymbol{n}}}^{\mathrm{(3)}}\otimes {{\boldsymbol{n}}}^{\mathrm{(3)}}$ where ${{\boldsymbol{n}}}^{\mathrm{(1)}}$ and ${{\boldsymbol{n}}}^{\mathrm{(2)}}$ are arbitrary transverse unit vectors forming an orthonormal basis with the void axis ***n***^(3)^. The internal parameters are related to porosity, which enters explicitly through the void volume fraction *f* and implicitly through *C*, *g*, $\kappa$, and *α*_2_, which are analytical functions of *f*, the aspect ratio of the void *w*, its orientation ${{\boldsymbol{n}}}^{\mathrm{(3)}}$, and eventually the components of . The principal directions of orthotropy of the matrix (i.e. of ), denoted L, T and S, are taken to be fixed relative to the material whereas the void orientation changes. Power-law hardening is assumed for the matrix: $\bar{\sigma }={\sigma }_{0}{(1+E\bar{\varepsilon }/{\sigma }_{0})}^{N}$ with *σ*_0_ the initial yield stress in tension along S and the components of  scaled accordingly. Also, *E* is Young’s modulus and *N* the hardening exponent. We also used 0.3 for Poisson’s ratio. The effective plastic strain $\bar{\varepsilon }$ evolves consistent with a plastic work equivalence principle^24^: $(1-f)\bar{\sigma }\dot{\bar{\varepsilon }}={\rm{\Lambda }}\sigma :\partial \, {\mathcal F} /\partial \sigma$ where ${\rm{\Lambda }}$ is the plastic multiplier. In the limit of a dense material (*f* = 0) with no penny-shaped cracks (*g* = 0), the yield condition ${\mathcal F} (\sigma )=0$ simply reads, in axes pointing onto the principal directions of :

2 $${\sigma }_{{\rm{H}}}^{2}\equiv \frac{3}{2}({h}_{{\rm{L}}}{s}_{{\rm{LL}}}^{2}+{h}_{{\rm{T}}}{s}_{{\rm{TT}}}^{2}+{h}_{{\rm{S}}}{s}_{{\rm{SS}}}^{2}+2{h}_{{\rm{LT}}}{s}_{{\rm{LT}}}^{2}+2{h}_{{\rm{TS}}}{s}_{{\rm{TS}}}^{2}+2{h}_{{\rm{SL}}}{s}_{{\rm{SL}}}^{2})={\bar{\sigma }}^{2}$$

where ${s}_{ij}={\sigma }_{ij}-1/3{\sigma }_{kk}{\delta }_{ij}$ are deviatoric stress components. All internal parameters evolve. The porosity rate is $\dot{f}=3{\rm{\Lambda }}(1-f)\partial \, {\mathcal F} /\partial {\sigma }_{kk}$ from plastic incompressibility of the matrix. The rate of change of void aspect ratio is given by:

3 $$\dot{w}/w=Q:[(1+{k}_{{\rm{w}}}{k}_{{\rm{f}}}{k}_{{\mathscr{T}}}){D}^{{\rm{p}}}+(\frac{1}{f}{X}^{v}-X){D}_{kk}^{{\rm{p}}}]$$

where *D*^p^ is the plastic strain rate associated with ${\mathcal F}$ and *X*^*v*^ is defined similar to *X* with *α*_2_ replaced by *α*_1_, a function of *f* and *w*. Also, *k*_w_, *k*_f_ and ${k}_{{\mathscr{T}}}$ are functions of *w*, *f* and the stress triaxiality ratio ${\mathscr{T}}$, calibrated on unit cell calculations. The voids are taken to rotate governed by Eshelby concentration tensors; see^43^ for details. Void coalescence by internal necking was checked using a micromechanical criterion^44^ resolved on a single coalescence system defined by normal ***e***_2_ along the *y* axis.

The above plastic relations were augmented with a hypoelastic law that employs the Jaumann rate of the stress within an objective co-rotational finite deformation framework. The nonlinear constitutive relations were integrated using a semi-implicit time integration scheme along with the Newton-Raphson method to solve the system of equations resulting from discretization of the constitutive equations. A user-defined material routine was developed within the commercial code ABAQUS. A consistent tangent matrix was employed in solving the force-displacement matrix equation for the global finite element problem. The solutions were obtained using structured meshes (~234000 degrees of freedom) that contained up to 18480 20-noded quadratic brick elements with reduced integration (eight Gauss points per element). The simulations were carried out on a quad processor Linux box at Texas A&M University.

## Supplementary information

Supplementary Information

Video 1

Video 2

Video 3

Video 4

Video 5

Video 6

Video 7

## Data Availability

All data is available in the manuscript or the supplementary materials.
