# Supplementary material for: Plastic flow anisotropy drives shear fracture
Source: Sci Rep. 2019 Feb 5;9:1425. doi: 10.1038/s41598-018-38437-y (PMC6363917; doi:10.1038/s41598-018-38437-y)
Supplement: Supplementary file 1 — Supplementary Information [file 41598_2018_38437_MOESM1_ESM.pdf]

Supplementary Information for

Plastic flow anisotropy drives shear fracture

A. Amine Benzerga, Nithin Thomas and Joshua S. Herrington

Correspondence to: benzerga@tamu.edu

This PDF file includes:

Supplementary Text
Figs. S1 to S4
Table S1
Captions for Movies S1 to S7

Other Supplementary Information for this manuscript include the following:

Movies S1 to S7

Supplementary Text

Summary of content

Here, we provide more details about the comparison with experiments, in particular the principle of estimating the values reported in Fig. 5 and the effect of full orthotropy. In addition, we illustrate details about the local effective response inside shear bands. Also, movies illustrate the process of shear banding with either plastic strain or void volume fraction distributions.

On comparison with experiments

Estimated anisotropy coefficients

Table S1 reports the set of Hill coefficients that best represent the 3D anisotropy of the three materials labeled T1, T2 and T3 in Fig. 5 of the paper. These coefficients were determined based on the plastic anisotropy ratio measurements reported by Basu et al.[1] for magnesium alloys. No claim is made about the suitability of Hill's criterion to represent accurately the more complex and evolving anisotropy of magnesium alloys. On far more accurate representations, the reader is referred to a recent article by our group[2]. The commonalty among findings in both experiments and simulations is that a variation in plastic anisotropy leads to a variation in the angle of shear fracture. Fig. S1 and Fig. S2 illustrate this fact in the simulations and experiments, respectively. The shear band angles extracted from these two figures are those reported in Fig. 5 of the main text demonstrating the strong effect of anisotropy on the shear band angle. In making comparisons in terms of shear band angles, we have chosen to use the values of shear coefficients h_{TS} and h_{SL} (Table S1) since these are hypothesized to determine the shear band angle. The spread of values in Fig. 5 corresponds to the difference in these two values for a given material.

Effect of full orthotropy

In order to gradually build understanding, we have focused on ideal forms of anisotropy whereby a single parameter, $h_{\mathrm{TS}}/h_{\mathrm{L}}$, determines the magnitude of anisotropy. A limited number of simulations were carried out with full orthotropic behavior for the matrix material, as the parameter space is now five-dimensional[1]. The values used for the Hill coefficients are listed in Table S1 under reference material 'R'. Any other set of values reported in Table S1 could have been used instead to convey the main point. Two misorientations were considered $\theta = 30°$, as in the main text, and $\theta = 45°$. Irrespective of misorientation and type of anisotropy, it was found that, if a shear band forms then it roughly lies in the same L–S plane. This further corroborates that shear coefficients h_{TS} and h_{SL} play a key role. For more general forms of orthotropy, it is possible that a change in the plane of shear banding occurs for other combinations of the Hill coefficients.

Interestingly, for $\theta = 45°$ no shear band was found to form for the transversely isotropic material. Figure S3a shows the load-displacement curves for both materials. The shear band does not form for the transversely isotropic case (Fig. S3b) because of symmetry, i.e., $h_{\mathrm{L}} = h_{\mathrm{S}}$ and $h_{\mathrm{TS}} = h_{\mathrm{SL}}$. In the plane of misorientation (L-S plane, see Fig. 1b) two nascent deformation bands tend to form but symmetry breaking is impossible. On the other hand, for the fully orthotropic matrix material 'R', all of the plastic anisotropy coefficients are different, in particular $h_{\mathrm{L}} \neq h_{\mathrm{S}}$, and this leads to symmetry breaking and shear banding, Fig. S3c.

Effective response in the shear band

Fig. S4a shows the evolution of Hill's equivalent stress, σ_{H}, given in units of the yield stress σ_0, for two points within the shear band. One point is at the core of the shear band and is labeled as being 'inside' while the other is sligthly away from the core but still plastically loading; it is labeled as 'outside' the core. The circle is a conservative indication of shear banding as detected by the onset of elastic unloading well outside the band.

The σ_{H} versus $\bar{\varepsilon}$ response is necessarily hardening, by definition. In order to get a better appreciation of the effect of shear banding on the effective response, Fig. S4b shows the evolution of the von Mises effective stress ($\sigma_{\mathrm{VM}}^2 = 3/2\, s_{ij} s_{ij}$) at corresponding locations. It can be seen that at incipient shear banding, the effective hardening rate is vanishingly small at some critical point but is positive everywhere else in the specimen.

[1] Only the ratios of any five of the six coefficients h_{XY} affect anisotropic yielding.

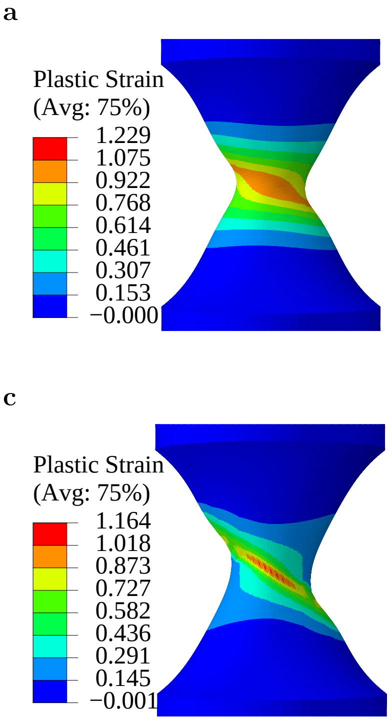

Figure S1. Simulated shear failure angles. Distributions of effective plastic strain after the onset of shear banding for an initial misorientation $\theta = 30^\circ$ using (**a**) $h_{\mathrm{TS}}/h_{\mathrm{L}} = 1.25$; (**b**) $h_{\mathrm{TS}}/h_{\mathrm{L}} = 1.5$; (**c**) $h_{\mathrm{TS}}/h_{\mathrm{L}} = 2.33$; (**d**) $h_{\mathrm{TS}}/h_{\mathrm{L}} = 5.0$. The shear band angles are reported in Fig. 5. All other simulation parameters are as in Fig. 1.

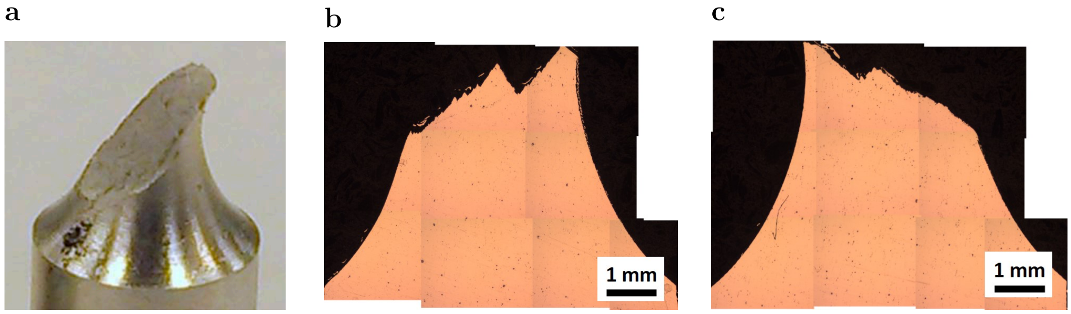

Figure S2. Experimental shear fracture angles in three anisotropic materials. Post-mortem measurement of average angles in the same type of round notched bar in (**a**) material T1, $\alpha_{sh} = 45 \pm 5°$; (**b**) T2, $\alpha_{sh} = 31 \pm 3°$; (**c**) T3, $\alpha_{sh} = 28 \pm 3°$. Corresponding coefficients of plastic anisotropy are given in Table S1.

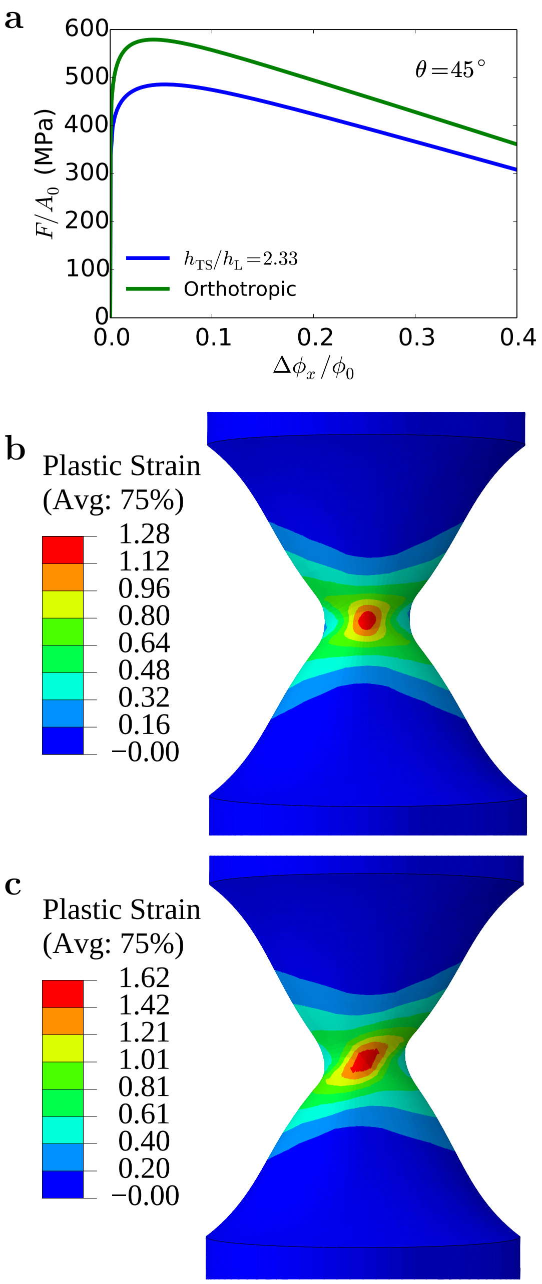

Figure S3. Analysis of the special case of intial misorientation $\theta = 45°$. (**a**) Load versus nominal strain curves for a transversely isotropic material with $h_{TS}/h_L = 2.33$ and orthotropic material 'R' (Table S1). (**b** to **c**) Distributions of effective plastic strain at the onset of void coalescence for (b) $h_{TS}/h_L = 2.33$ and (c) material 'R'. All other simulation parameters are as in Fig. 1.

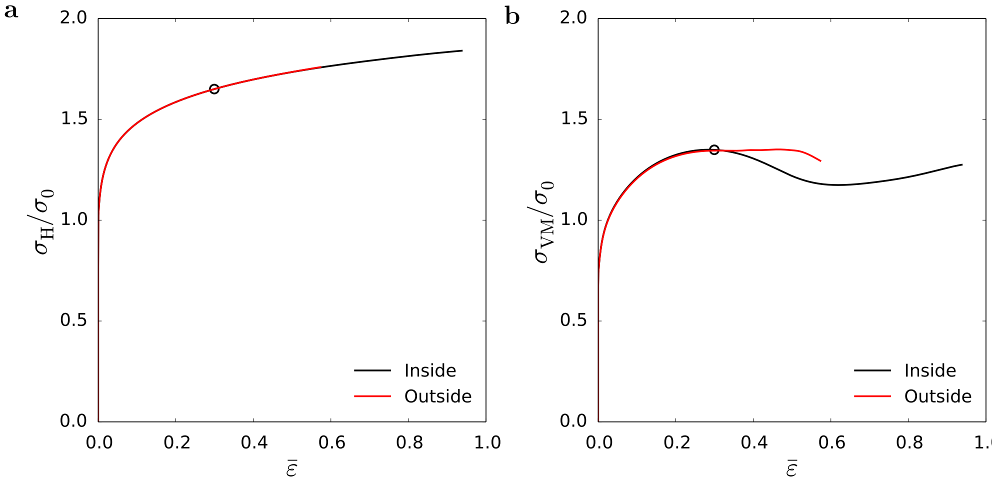

Figure S4. Effective behavior in the shear band. (**a**) Hill's equivalent stress, σ_H in Eq. (2) versus effective plastic strain, $\bar{\varepsilon}$, at two points inside the shear band, one inside and one outside the core of the shear band. (**b**) The von Mises equivalent stress σ_{VM} versus $\bar{\varepsilon}$. The circle indicates the onset of the shear band. Simulation parameters as in Fig. 1.

Table S1. Estimated values of plastic anisotropy coefficients identified in tension for the three materials labeled T1–T3 studied by Basu et al.[1]. The nomenclature (S, L, T) matches theirs as (E, L, F). All values are normalized by h_{L}. Data for reference material 'R' are taken from the literature[3].

Hill Coefficient	T1	T2	T3	R
h_{S}	6.1	5.0	3.2	0.4
h_{L}	1.0	1.0	1.0	1.2
h_{T}	3.9	3.2	2.4	0.9
h_{LT}	3.2	3.0	6.7	1.0
h_{TS}	1.7	1.5	1.4	1.8
h_{SL}	2.8	2.6	2.1	1.6

Movie S1.
The movie shows the deformation of the full bar with superposed contours of effective plastic strain $\bar{\varepsilon}$. Only the lateral surface is visible but the pattern is similar in a meridian section in the x–y plane, as the shear band extends through the bar. The movie shows how the top surface moves slightly right when the transient deformation band develops then moves back to the left when a shear band of opposite sign forms and develops. Lateral motions are induced and not imposed. The only displacement imposed to the top surface is vertical. The snapshots shown in Fig. 1 are extracted from this simulation using $\theta = 30^\circ$ and $h_{\mathrm{TS}}/h_{\mathrm{L}} = 2.333$.

Movie S2.
The movie shows contours of the void volume fraction, or porosity f. Only the lateral surface is visible. Throughout the deformation, the porosity remains small until the very last stages when it develops on the outer surface showing a tearing-like incipient fracture. The calculation was terminated when void coalescence was detected inside the bar, as shown in the following movie.

Movie S3.
The movie shows the exact same simulation as in the four previous movies, but here the porosity contours are shown in a meridian section (x–y plane). Due to higher hydrostatic stress at the center of the bar, the porosity develops much faster there than it does near the free surfaces in Movie S2.

Movie S4.
The movie shows the process of shear banding in a bar with initial misorientation $\theta = 30^\circ$ using $h_{\mathrm{TS}}/h_{\mathrm{L}} = 1.5$ (case reported in Fig. 5 and shown in Fig. S1b). The contours are those of effective plastic strain $\bar{\varepsilon}$.

Movie S5.
The movie shows the process of shear banding in a bar with initial misorientation $\theta = 30^\circ$ using $h_{\mathrm{TS}}/h_{\mathrm{L}} = 5.0$ (case reported in Fig. 5 and shown in Fig. S1d). The contours are those of effective plastic strain $\bar{\varepsilon}$.

Movie S6.
The movie illustrates what happens in the case $h_{\mathrm{TS}}/h_{\mathrm{L}} < 1$, as discussed in conjunction with Fig. 5. Here, $h_{\mathrm{TS}}/h_{\mathrm{L}} = 0.8$. All other simulation parameters are the same as in Fig. 1 and previous movies. Here, contours of effective plastic strain are shown inside the bar (meridian x–y section). The deformation band inclined at 30° does not form until very large strains. By then, void coalescence occurs at the center of the bar and the simulation is terminated. The bar proved very stiff to forming a shear band.

Movie S7.
This movie shows contours of porosity that go along with the previous movie (inside the bar). The bar deforms symmetrically in the x–y plane and the porosity develops faster at the center due to high stress triaxiality. No shear band was found to form.

References

1. Basu, S., Dogan, E., Kondori, B., Karaman, I. & Benzerga, A. A. Towards Designing Anisotropy for Ductility Enhancement: A Theory-Driven Investigation in Mg-alloys. *Acta Materialia* **131**, 349–362 (2017).
2. Kondori, B., Madi, Y., Besson, J. & Benzerga, A. A. Evolution of the 3D plastic anisotropy of HCP metals: experiments and modeling. *Int. J. Plast.* (2018). DOI 10.1016/j.ijplas.2017.12.002. In press.
3. Kondori, B. & Benzerga, A. A. Fracture strains, damage mechanisms and anisotropy in a magnesium alloy across a range of stress triaxialities. *Exp. Mech.* **54**, 493–499 (2014).
